# Application of Dendrimers in Anticancer Diagnostics and Therapy

**DOI:** 10.3390/molecules27103237

**Published:** 2022-05-18

**Authors:** Zuzanna Bober, Dorota Bartusik-Aebisher, David Aebisher

**Affiliations:** 1 Department of Photomedicine and Physical Chemistry, Medical College of Rzeszów University, 35-310 Rzeszów, Poland; zbober@ur.edu.pl; 2 Department of Biochemistry and General Chemistry, Medical College of Rzeszów University, 35-310 Rzeszów, Poland; dbartusikaebisher@ur.edu.pl

**Keywords:** PAMAM dendrimers, targeted drug delivery, theranostics, MRI

## Abstract

The application of dendrimeric constructs in medical diagnostics and therapeutics is increasing. Dendrimers have attracted attention due to their compact, spherical three-dimensional structures with surfaces that can be modified by the attachment of various drugs, hydrophilic or hydrophobic groups, or reporter molecules. In the literature, many modified dendrimer systems with various applications have been reported, including drug and gene delivery systems, biosensors, bioimaging contrast agents, tissue engineering, and therapeutic agents. Dendrimers are used for the delivery of macromolecules, miRNAs, siRNAs, and many other various biomedical applications, and they are ideal carriers for bioactive molecules. In addition, the conjugation of dendrimers with antibodies, proteins, and peptides allows for the design of vaccines with highly specific and predictable properties, and the role of dendrimers as carrier systems for vaccine antigens is increasing. In this work, we will focus on a review of the use of dendrimers in cancer diagnostics and therapy. Dendrimer-based nanosystems for drug delivery are commonly based on polyamidoamine dendrimers (PAMAM) that can be modified with drugs and contrast agents. Moreover, dendrimers can be successfully used as conjugates that deliver several substances simultaneously. The potential to develop dendrimers with multifunctional abilities has served as an impetus for the design of new molecular platforms for medical diagnostics and therapeutics.

## 1. Introduction

**Figure 1:**
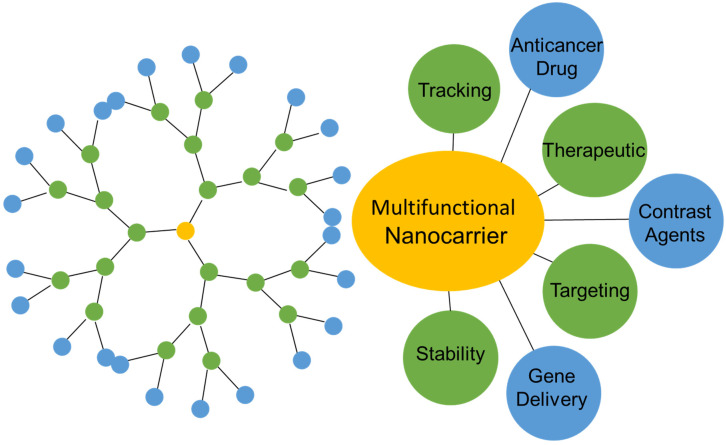
Dendrimers applications.

The potential applications of dendrimers in many fields of science and medicine are vast [1]. Recently, there have been a number of reports in the literature that have demonstrated the potential of using dendrimers, inter alia, as theranostic agents and in genetic engineering. There has been intense interest in the development of dendrimers as carriers for pharmaceutical substances. Moreover, research on the use of dendrimers as MRI contrast agents is of interest since dendrimer-based contrast agents shorten relaxation times and improve contrast and pharmacokinetics simultaneously. The standard contrast used for MRI examination contains gadolinium (Gd^3+^) complexes with organic chelating acids, such as DOTA (1,4,7,10-tetrazacyclodecyl-1,4,7,10-tetraacetic acid) or DTPA (diethylenetriaminepentaacetic acid) [1], which, in their pure forms, are highly toxic and accumulate in the liver and bones [2]. The introduction of dendrimer-based contrast agents or therapeutic substances for clinical applications is associated with a determination of the safety of use as well as the verification of their toxicity and biocompatibility. Due to the fact that dendrimers are highly modifiable molecules (Figure 1), their potential as safe drug carriers and contrast agents has been explored [3].

**Figure 2:**
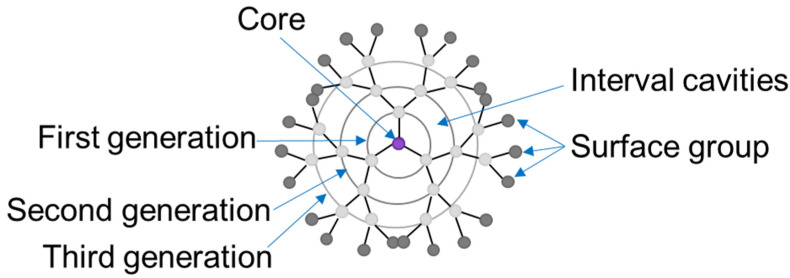
Diagram of a dendrimer structure.

Dendrimers are characterized by a symmetrical, highly branched three-dimensional structure with a strictly defined molecular weight [4]. They are similar in structure to a sphere. The structural feature consists of a core from which dendrons radiate (Figure 2). The terminal ends of dendrons can be covalently or non-covalently attached to biologically active molecules, drugs, genes, contrast agents, and other reporter groups [5].

**Figure 3:**
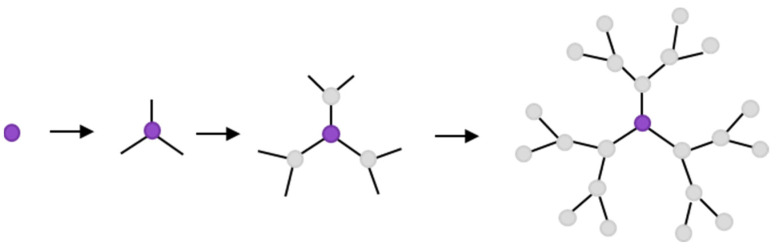
The divergent method of dendrimer synthesis.

Dendrimers that have terminal amine groups (-NH_2_) or hydroxyl groups (-OH) are termed complete dendrimers, while those that terminate with carboxyl groups are termed half-dendrimers [6]. Depending on the dendrimer generation, the packing density of the molecules in the surface region increases, which inhibits their further growth [7]. As a result, dendrimer molecules can only be synthesized up to generation 10. Regardless of how dendrimers are synthesized, the method of making dendrimers is based on a repeated sequence of several reactions. Each duplication results in the synthesis of an increasingly higher generation of the dendrimer structure. In addition, each successive generation benefits from doubling the number of active residues on the surface of the molecule. The synthesis of dendrimers makes it possible to control each stage of generation, resulting in a strictly defined structure [8]. There are various types of dendrimers, including phosphorus (P-dendrimers) [9], polyamidoamines (PAMAM) [10], polyamines [11], polyamides [12], polypeptides [13], polyesters [14], carbosilicates (CBS) [15], poly(L-lysine) dendrimers (PLL) [16], polyesters (PGLSA-OH) [17], poly (2,2) acid dendrimers -bis (hydroxymethyl) propionic (bis-MPA) [18], and peptide dendrimers [19]. In addition, we also distinguish dendrimers based on sugar units and oligonucleotides. The most frequently described dendrimers in the literature are PAMAM dendrimers and polypropyleneimine (PPI) dendrimers. For instance, recent reports have described the interaction between rose bengal and cationic PAMAM and PPI dendrimers of the third and fourth generation [20]. One method of synthesizing dendrimers is the divergent method. The preferred dendrimer is formed by the attachment of successive monomer layers to the dendrimer core (Figure 3).

**Figure 4:**
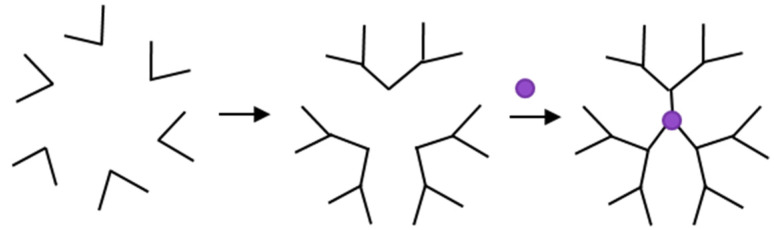
The convergent method of dendrimer synthesis.

The divergent, synthetic method of dendrimer synthesis causes a formation of successive layers of monomers, resulting in the increasing branching of the dendrimer arms. The above fact may result in the loss of reactivity of terminal functional groups. The second method of synthesizing dendrimers is the convergent method [8]. In this method, the first step involves the synthesis of the branched arms of the dendrimers, while the second step of the synthesis involves joining them with a multifunctional core. A schematic presentation of the convergent method of dendrimers is shown below (Figure 4). The use of this method allows for greater control during the synthesis, resulting in products that are free from defects.

## 2. Different Generations of Polyamidoamine (PAMAM) Dendrimers

**Figure 5:**
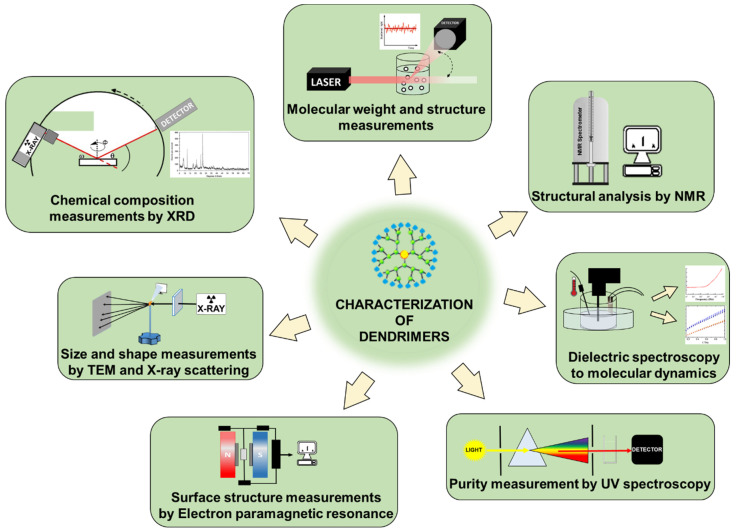
Various analytical techniques for the characterization of dendrimers.

Commonly used dendrimers that are either synthesized or obtained commercially are polyamidoamine type cationic dendrimers (PAMAMs), which can be formed up to 10 generations. A characteristic feature of PAMAM dendrimers is their structure, which consists of a core in the form of ammonia or ethylenediamine (EDA) and attached molecules of methyl acrylate and ethylenediamine. Due to their specific, characteristic structure with regions of free spaces (cavities), it is possible to use them as carriers for anti-cancer drugs, gene transfection, or non-proton imaging. Depending on the generation of dendrimers, their size increases. The size of the dendrimers will be related to the packing density of the molecules on the surface of the dendrimer. Tomalia et al. described a linear increase in diameter and an exponential increase in the number of surface groups for PAMAM dendrimers from the core to the seventh generation (G2–G7), with diameters of G2 = 2.0 nm, G3 = 3.1 nm, G4 = 4.0 nm, G5 = 5.3 nm, G6 = 6.7 nm, and G7 = 8.0 nm [21]. The synthesis of dendrimers requires the implementation of chromatography and mass spectrometry for characterization. Basic analytical methods also include NMR, optical activity (circular dichroism), purity (UV spectroscopy and Electrophoriesis), surface structure (Electron paramagnetic resonance), size and shape measurements (TEM, X-ray), and X-ray crystallization (XRD) (Figure 5).

## 3. The Use of Dendrimers in Drug Delivery

The properties of dendrimers are used as carriers of pharmacological compounds. When the drug is confined in the cavities of the dendrimer, encapsulation is performed so that a sustained release conjugate can be obtained. Increasingly, dendrimers are used as carriers for pharmaceutical substances [22], which can be placed in two ways—either on the surface of the dendrimer, or inside it by encapsulation, i.e., enclosing the molecules in the cavities of the dendrimers. In the case of the structure of the three-dimensional dendrimers, it is possible to use a wide variety of drugs forming biologically active drug conjugates. Due to the penetration of dendrimers through the cell membrane, they are beginning to be used as non-viral gene transporters. Dendrimers can be used to create new generation vaccines. Currently, nanotechnology and combination therapy are the main promising areas in the fight against cancer. The implementation of dendrimers can contribute to the improvement of the anti-cancer therapies [23,24]. In the latest work by Kaczorowska et al., the modification of PAMAM G3 by attaching biotin via an amide bond and glucoheptoamidated by adding α-D-glucoheptone-1,4-lactone is reported. A series of conjugates with a variable number of biotin residues was obtained, while reactive oxygen species were detected in Caco-2 cells incubated with capsules after 30 s of irradiation with a 655 nm laser beam [25]. On the other hand, the group of Salimi et al. presented the use of dendrimer functionalized with iron oxide nanoparticles (G4 @IONPs) in the therapy of magnetic hyperthermia of breast cancer in Bagg albino C (BALB/c) mice. It has been shown to reduce the viability of cancer cells and inhibit tumor growth, which indicates the possibility of using dendrimers as nanoparticles for therapeutic applications [26]. In the literature, we can also find many reports on the use of dendrimers in the treatment of breast cancer [27]. Kulhari et al. presented PAMAM dendrimers conjugated with Trastuzumab to improve the delivery of docetaxel to breast cancer cells overexpressing the HER2+ receptor. Conjugated dendrimers showed higher cellular internalization and the induction of apoptosis against MDA-MB-453 cells [28]. On the other hand, Aleanizy et al. described the use of nanocapsules containing G4 PAMAM polyamidoamine dendrimer with neratinib and attached to trastuzumab, which was characterized by increased cellular uptake in HER2+ breast cancer cells [29]. In order to improve the efficiency of HER2+ tumor detection, new nanoimaging agents were developed using PAMAM G5 dendrimers, gold (AuNP), and gadolinium (Gd) nanoparticles conjugated to a humanized anti-HER-2 antibody (Herceptin). The obtained results show that it is possible to use dendrimers as nanodiagnostics or nanotherapeutic agents in the treatment of HER2+ tumors [30]. In addition, G5 PAMAM dendrimers hydrophobically modified by lipid-like myristic acid (My) tail grafting enhance delivery of tamoxifen to breast cancer cells in in vitro studies [31]. Studies also show a glucose-modified PAMAM dendrimer used to deliver doxorubicin to breast cancer cells, which increases the cytotoxic activity of the conjugate in MCF-7 breast cancer cells [32]. A herceptin-targeted PAMAM G4 dendrimer functionalized with diglycolamic acid (DGA) was also used as a drug carrier for cisplatin in human ovarian cancer HER-2+ and HER-2− [33]. Reports also show that poly (ε-caprolactone) (PCL)-poly (ethylene glycol) (PEG) (PEG-PCL NP) nanoparticles, combined with Trastuzumab, can be used for the targeted therapy of gastric cancer cells with HER2+ receptor overexpression [34]. Moreover, other studies show the possibility of using dendrimers as a carrier coupled with a photosensitizer for photodynamic therapy as a targeted delivery of photosensitizers to neoplastic tissue [35].

### 3.1. Dendrimer Conjugates with Pharmaceutically Active Substances In Vitro

Dendrimers have unique properties; their unique structure allows them to be used as carriers for pharmaceutical substances. Numerous in vitro studies on pharmaceutical substances show their great potential. The use of dendrimers for drug delivery can be achieved in two ways. One is drug-polymer conjugation. In the case of hydrophobic drugs, they can be placed inside the hydrophobic interior of the dendrimer. Then, they have very good solubility in water. The second way is by covalent coupling on the surface of the dendrimer. It should be noted that amine terminated dendrimers may cause toxicity by binding to negatively charged cell membranes. Targeted drug delivery in anti-cancer therapy can bring many benefits, as the drug goes directly to the neoplastic lesion. Table 1 provides an overview of the various dendrimer conjugates with pharmaceutically active substances.

### 3.2. Application of Dendrimers in Oncological Therapy

Dendrimers are characterized by unique structural properties and a specific shape. Dendrimers can be successfully used in cancer therapies as drug carriers due to covalent conjugation or by physical encapsulation [48]. In addition, dendrimers are increasingly used in oncological therapies. They accumulate directly in the tumor, which ensures the delivery of the drug in the prescribed dose. In the case of traditional chemotherapy, the problem turns out to be low efficiency in the distribution of drugs to the neoplastic tissue [49]. Additionally, it enables an increase in the effectiveness of the treatment and also helps to reduce cytotoxicity to normal cells. The implementation of the use of dendrimers in oncological therapies enables the targeted delivery of drugs in an effective dose and the monitoring of the effectiveness of anticancer therapy. An increasingly frequent problem is the inability to verify the effectiveness of the therapies used, or the fact that the ability to check the effectiveness of the therapies is largely limited. Another problem is the drug resistance and cytotoxicity to normal cells, which is related to the lack of specificity for neoplastic cells. The use of dendrimers allows for the neutralization of the problems encountered during the application of anti-cancer therapies. Currently, numerous studies are being carried out on their use as carriers of therapeutic molecules. The multifunctional core and the branching units are terminated with free functional groups. Depending on the chemical structure of the core, its branches and functional groups and their reactivity, size, and shape vary [50]. Due to the specificity of the construction, various types of modifications are possible. For this reason, it is possible to use them in targeted therapies, because, by attaching ligands, they bind directly to the receptors in neoplastic tumors [51]. This allows the drug to penetrate inside the neoplastic cells, avoiding the increasing phenomenon of resistance to therapeutic agents [52]. The use of dendrimers as drug carriers and their use in targeted therapy allows for better effectiveness of the applied therapies and many side effects. [53]. PAMAM dendrimers can increase the solubility of hydrophobic drugs and can be used in targeted therapy [54,55,56]. Additionally, polyphenylene dendrimers directly targeting the tumor and pH responsive release in tumors are a promising nanocarrier for anti-cancer drugs due to their improved drug encapsulation properties [57]. A polypropylene dendrimer is used as a carrier for pharmaceutical substances such as anti-cancer drugs, as well as genes [58]. The development of carriers that will influence the shedding of oncogenes and cancer-related genes is extremely important in the treatment of cancer. The dendrimer architecture and the influence of the core have a significant influence on the effectiveness and biocompatibility of the therapies used [59]. Dendrimers are also used to deliver siRNA and DNA. However, designing this type of particles in an efficient and biocompatible manner to deliver siRNA or DNA is a very big challenge for researchers. Due to their properties, dendrimers can be carriers of bioactive molecules. The modification of dendrimers allowed for the development of a gene delivery vehicle by modifying the compound containing guanidyl groups and phenyl groups on the surface of the cationic dendrimer [60]. The potential of using poly (propylene-imine) dendrimers (PPI) as a vehicle for the docetaxel (DTX) targeting of MCF-7 breast cancer was assessed by Thakur [61]. The synthesis of PPI dendrimers with the drug gemcitabine has also been used in the targeted therapy of A549 lung cancer cells [62]. PPI dendrimers have been used to increase paclitaxel (PTX) delivery to the brain [63]. Other studies have used a transferrin-containing dendrimer for the targeted therapy of the brain [64]. Fourth generation PPI dendrimers partially modified with maltose were used as carriers for the CCRF-1301 lymphoid leukemia cell lines and the HL-60 myeloid cell line [65]. Additionally, Franiak-Pietryga presented in vivo studies on the use of maltotriose-modified PPI dendrimers as a potential new platform in the treatment of chronic lymphocytic leukemia [66].

### 3.3. Use of Dendrimers as Contrast Agents

**Figure 6:**
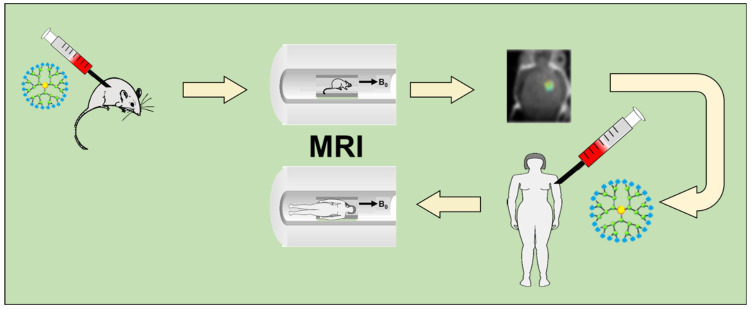
Application of MRI.

Contrast agents used in magnetic resonance imaging (MRI) allow for the obtention of more sensitive images. The two common types of images are hyperintense images (T_1_ weighted images) and hypointense images (T_2_ weighted images). On the other hand, the use of contrast agents allows for the shortening of the longitudinal relaxation time, which is used in clinical diagnostics and in scientific research (Figure 6). The most commonly used contrast agents in clinical practice contain paramagnetic gadolinium compounds (Gd^3+^). However, due to the occurring side effects, research is underway on a new generation of Gd-MRI contrast agents, which could be administered in lower doses while ensuring high sensitivity. Therefore, in scientific publications, we can follow more and more reports on Gd-functionalized dendrimers. Gadolinium-based contrast associated with dendrimers increases the number of Gd^3+^ in the molecule, which makes them more spherical and in turn increases their sensitivity [67]. Moreover, attention should be paid to the accumulation of contrast agents in the neoplastic tissue. In the case of multiparticulate contrast agents, they should tend to target neoplastic tissues much more than healthy tissues, due to the undeveloped lymphatic vessels in the tumor that would eliminate these particles. In studies, we can find reports on the use of different types of dendrimers as MRI contrasts [68]. They are increasingly used as contrast agents for MRI. Dendrimers are woody macromolecules with numerous chemical groups with which Gd chelates can be combined. Moreover, we can control their size and nanoscopic dimension. The most popular are metal salts showing paramagnetic properties, such as (Gd (III)-DOTA) and its derivatives. Studies show numerous uses of such agents in combination with polysaccharides, polyamino acids, or proteins used for imaging animal models, which could be used in clinical imaging at a later stage. A new MRI contrast mechanism, i.e., chemical saturation transfer by chemical exchange (CEST), is also presented. Lesniak et al. used nanocarriers based on G5 PAMAM dendrimers with covalently bonded salicylic acid to the surface of the dendrimer. It has been shown that the conjugate can be used for imaging gliomas in mice, as a CEST contrast of 9.4 ppm was obtained [69]. Additionally, the group of Snoussi et al. presented the sensitive CEST agents based on the exchange of nucleic acid iminoprotons: the detection of poly (rU) and the dendrimer-poly (rU) model [70]. On the other hand, the group of Shen et al. has developed a composite of polylysine dendrimer and Fe_3_O_4_ coupled to a heterogeneous dimer with a peptide acting as a probe for the early diagnosis and treatment of hepatocellular carcinoma in order to reduce the adverse effects caused by doxorubicin (DOX). MRI studies have shown that MNP-DGL-RGD-GX1-DOX can be used for the early diagnosis and therapy of hepatocellular carcinoma [71]. By contrast, Zhu et al. presented the Mn (II) chelating dendrimer as an MRI contrast agent containing six tyrosine derivatives of the [Mn (EDTA) (H_2_O)]2− group linked to the cyclotriphosphazene core. It showed great relaxation, which confirms that Mn (II) is a potential alternative to Gd-based measures [72].

### 3.4. In Vitro Study—Use of Dendrimers As Contrast Agents

The use of dendrimers allows for the preparation of macromolecular contrast agents used for diagnostic tests. Contrast agents based on dendrimers significantly improve contrast properties and improve pharmacokinetic properties. The use of dendrimers as contrast media carriers has gained popularity in recent years. They are used, inter alia, for the diagnosis of nuclear magnetic resonance. Table 2 below provides an overview of the different types of dendrimers used as contrast agents in in vitro testing.

### 3.5. In Vivo Study—Use of Dendrimers As Contrast Agents

Due to the prevalence of neoplastic diseases, it is important to develop in vivo research in the field of MR imaging. MRI diagnostics have developed significantly in recent decades, but due to civilization changes and the relatively late detection of neoplastic changes, there is a need for further development in this field. The use of nanoparticles in clinical diagnostics would enable the early detection of tumors and thus the implementation of treatment at an earlier stage. They are increasingly used as contrast agents for MRI diagnostics. Nanoparticles are characterized by a small particle size and the ability to accumulate in the tumor area, thus improving the efficiency of imaging. On the other hand, multifunctional nanoparticles can be used for simultaneous diagnostics and therapy. The use of nanoparticles allows for better image contrast, which enables better visualization of pathologically changed structures. Gadolinium-based contrast agents are the most commonly used in clinical diagnostics. The combination of Gd molecules with dendrimers allows for the obtention of images of greater sensitivity due to increased accumulation in the tumor area. For example, in vivo MR imaging of atherosclerosis manganese dendrimer G8 was used, which was conjugated with MnDTPA (ion 758 Mn) and the MDA2 antibody targeting malondialdehyde (MDA) -lysine epitopes to significantly enhance atherosclerotic lesions [99]. In their research, Chen et al. presented a G5 PAMAM dendrimer conjugate encapsulated with gold nanoparticles chelated with gadolinium and anti-human HER2 antibody, which increased MRI signal intensity by approximately 20% in the mouse HER2+ tumor model [100]. Additionally, Gonawala et al. used the PAMAM G5 in an animal model of glioblastoma [101]. The research also presents a fourth-generation biodegradable dendritic contrast agent (DCA) that can be successfully used as a contrast agent for imaging liver metastases by MRI, which has been hampered by the accumulation of contrast agents in hepatocytes and Kupffer cells. DCA, on the other hand, reduces the background signal in the liver, thereby enhancing the MRI signal of tumors [102]. Other studies presented a G3 dendrimer synthesized with folic acid. In vivo studies confirmed that the use of such a modification enables the obtention of a clear SPECT image, which may contribute to the improvement of cancer diagnostics [103]. Reports also show the popular Magnevist counter agent attached to the G1 spherical dendrimer, which resulted in increased tissue relaxation and improved MR image contrast [104].

Table 3 below provides an overview of the different types of dendrimers used as contrast agents in in vivo studies.

## 4. Summary

The development of nanotechnology is an innovative approach to replace the classic regimens of anti-cancer therapies and classic diagnostics. The modification of drugs and contrast agents that already exist, as well as the search for new ones, is the future and a challenge of modern medicine. The use of dendrimer-coated magnetic nanoparticles allows for the development of a drug delivery system and targeted therapy that improves the effectiveness of therapy and reduces the risk of toxicity by using a lower drug dose. In addition, implementing treatment regimens using pH-sensitive pharmaceuticals would help to reduce drug resistance during anti-cancer therapy. Therefore, it is essential to characterize dendrimers and pay attention to analytical techniques in the process of their manufacture. Depending on the dendrimer group and the surface group, it is possible to create conjugates of interest. The use of nanoparticles allows for the minimization of side effects and the adjustment of the appropriate dose of the drug, which will bring the desired therapeutic effect. Due to their properties and the ability to adjust the size of molecules, PAMAM dendrimers, used as drug carriers, can be successfully used in anti-cancer therapy. It is possible to attach specific ligands to the surface of the dendrimer, which will be recognized by receptors overexpressing cancer cells. In addition, research into new MRI contrast agents is proving to be important. The currently used contrast agents cause a number of side effects due to the high dose administered. The research focuses on the development of contrast agents based on functionalized Gd dendrimers that could be administered in clinical trials at a lower dose while maintaining the same sensitivity and diagnostics in clinical trials as traditional contrast agents. This review presents advancements in dendrimer-based research in various fields. It should be emphasized that the spectrum of the use of dendrimers is very wide. The applications of dendrimers include, but are not limited to, chemotherapy, radiation therapy, photothermia, and photodynamic therapy. In addition, they are also used in gene therapies because they allow for the condensation of nucleic acid materials. Due to the unknown metabolism of the therapies used, there is a need for further research into the use of dendrimers, despite the fact that they are characterized by higher biocompatibility and the improved effectiveness of the therapies used. Despite numerous studies, there is a need for continued research and an attempt to transfer them to in vivo research. There is a need to improve the effectiveness of the applied therapies and to understand metabolic mechanisms. In summary, the use of dendrimers in oncology is a modern approach that can overcome the limitations of standard diagnostics and therapy by improving the time required to detect neoplastic lesions and monitoring the effectiveness of the therapy. The use of dendrimers for the synthesis of new drugs or the modifications of existing drugs is a challenge of modern pharmacology. In addition, the development of effective drug delivery methods to cancer cells would allow for better therapy outcomes. The development of targeted therapies would make it possible to improve the effectiveness of treatment and minimize the negative side effects of the used therapies. It will also make it possible to bypass the disadvantages of classical diagnostic and therapeutic methods and improve the effectiveness of the used therapies.

## Figures and Tables

**Table 1:** Use of various dendrimer conjugates with pharmaceutically active substances.

Surname, Year	Type of Dendrimer/Fluorination	Application and Results	Ref.
Wang et al., 2021	Nano-in-Nano Dendrimer Gel Particles with brimonidine tartrate (BT) and timolol maleate (TM)	Efficient topical delivery of antiglaucoma drugs into the eye.	[36]
Bartusik-Aebisher et al., 2021	Trastuzumab-dendrimer-fluorine	To treat breast cancer cells in vitro, monitored by MRI measurements.	[37]
Mekonnen et al., 2019	Gadolinium ferrite nanoparticle in generation 4.5 poly(amidoamine) dendrimer	G4.5-GdIO is a promising alternative non-invasive MRI-tracked anti-cancer drug delivery system.	[38]
Marcinkowska et al., 2018	Conjugate of PAMAM Dendrimer, Doxorubicin, and Monoclonal Antibody—Trastuzumab:	Use in HER-2 positive (SKBR-3) and negative (MCF-7) human breast cancer cell lines.	[39]
Mirzaei et al., 2015	Anionic linear globular dendrimer (ALGDG2)	Use of nanoconjugate in 1H-NMR imaging and ^17^ O-NMR in in vitro studies.	[40]
Jain et al., 2015	G5 conjugated with Muramyl dipeptide and with Amphotericin B	There was a significant reduction in toxicity in the haemolytic toxicity and cytotoxicity studies in R774A.1 erythrocytes and macrophage cells.	[41]
Marcinkowska et al., 2015	PAMAM dendrimer-trastuzumab conjugates that contain docetaxel or paclitaxel	It was shown to be highly toxic to SKBR-3 HER-2 positive cells and lowly toxic to MCF-7 HER-2 negative cells.	[42]
Ma et al., 2015	TMAB-poliamidoamina (PAMAM) with paklitaksel (PTX)	PAMAM conjugated to TMAB was taken up by BT474 cells overexpressing HER-2 more efficiently than MCF-7 cells, which expressed lower levels of HER-2.	[43]
Leng et al., 2013	G4 conjugated with chitosan I methotrexate nanoparticle	Improvement of the cytotoxicity of free methotrexate on cells.	[44]
Rouhollah et al., 2013	G2, G3, G4, and G7 dendrimers conjugated with magnetic nanoparticles (Fe_3_O_4_) and DOX	G4 Fe_3_O_4_ dendrimer releases most of the drug at a lower pH, proving to be the most acceptable generation for effective DOX delivery.	[45]
Mullen et al., 2010	G5 PAMAM dendrimer	In this study, the amide coupling methods commonly used to conjugate ligands to poly(amidoamine) (PAMAM) dendrimers were examined.	[46]
Wang et al., 2003	G3-Polihydroksyalkanian/Tamsulosin	Drug solubility is purported to be improved by amine-terminated dendrimers.	[47]

**Table 2:** Use of dendrimers as contrast agents in in vitro studies.

Surname, Year	Type of Dendrimer/Fluorination	Application and Results	Ref
Song et al., 2021	Core-shell tecto dendrimers	MRI through an amplified passive EPR effect and also further extended for different cancer theranostic applications.	[73]
Zhang et al., 2020	OEG Gn-PROXYL radical dendrimers	Used as contrast agents in MRI	[74]
Shrestha et al., 2020	Gadolinium complexes attached to poly ethoxy ethyl glycinamide (PEE-G) dendrons	As contrast agents with enhanced relaxation in MRI	[75]
Hectors et al., 2018	G5 dendrimer, G2 dendrimer	Multiagent DCE-MRI (combination of contrast agents and low and high molecular weight) to improve the accuracy of the assessment of tumor hemodynamic parameters and vascular permeability; sequential injection of G5 dendrimer, G2 dendrimer, and Gd-DOTA	[76]
Kondo et al., 2017	Chiral dendrimer (S-isomeric dendrimer)	Chiral dendrimer Gd-MRI CAs, which showed high r1 values; association constant values (Ka) of S-isomeric dendrimer CAs to bovine serum albumin (BSA) were higher than those of R-isomeric dendrimer CAs (contrast agents)	[77]
Luong et al., 2017	Folic acid-polyamidoamine dendrimers surface (FA-PAMAM)	Polyvalent theranostic nanocarrier consisting of superparamagnetic iron oxide nanoparticle core (SPIONs) decorated with folic acid-polyamidoamine dendrimers surface (FA-PAMAM); research on the overexpression of ovarian (SKOV3) and cervical (HeLa) cells	[78]
Gündüz et al., 2016	Poly(amidoamine) (PAMAM) dendrimers	MRI contrast agents; CA provides a longer tissue retention time due to its high molecular weight and size	[79]
Gündüz et al., 2016	Generation 4 (G4) poly-(amidoamine)(PAMAM) dendrimer	They developed a nanosized, calcium-sensitive dendrimeric probe that changes longitudinal and transverse relaxation times	[80]
Haribabu et al., 2016	3G polyamidoamide (PAMAM) dendrimers	MRI contrast agents, dual mode (T_1_ and T_2_) contrast agent based on folic acid functionalized manganese ferrite nanoparticles (MNP) entrapped in 3G polyamidoamide (PAMAM) dendrimers;	[81]
Miyake et al., 2015	1st, 2nd, and 3rd-generation chiral dendrimer-triamine- coordinated CAs	MRI contrast agents (Gd-MRI CAs), which showed longitudinal relaxivity (r_1_) values	[82]
Huang et al., 2015	Paramagnetic dendrimers up to the fourth generation (i.e., G1-G4); poly(amido amine) (PAMAM)	Create a dual-modality nanosized contrast agent	[83]
Malone et al., 2015	5th-generation PAMAM dendrimer	Cell-penetrating peptides and their Gd-loaded dendrimeric form (ACPPD-Gd) have been shown to selectively accumulate in tumors	[84]
Bhuiyan et al., 2015	G5-PAMAM dendrimer	The MRI contrast agent nanoprobe (GdDOTA-4AmP)44-G5, at 3T and 7T magnetic field strengths, shows pH response in the range commonly found in the microenvironment of solid tumors	[85]
Cai et al., 2015	Multilayers of poly(γ-glutamic acid) (PGA)/poly(L-lysine)/PGA/folic acid (FA)-modified dendrimer	Efficient nanoprobe for the targeted dual mode CT/MR imaging of a xenografted tumor model	[86]
Yu et al., 2015	1st-generation dendron (G1-OH); dendrons 3, 10, 12, 14	The dendrimer is characterized by a strong ^19^F NMR peak and short relaxation times	[87]
Wang et al., 2014	Bimodal nanoprobe	Quantitative ^19^F MRI and NIR fluorescence bioimaging and cell tracking	[88]
Filippi et al., 2014	Amphiphilic Janus dendrimers (dendrimersomes)	Efficient and versatile nanoplatform for biomedical imaging	[89]
Ghalandarlaki et al., 2014	Dendrimer-G1	New nano contrast medium increases its effectiveness	[90]
Lee and Ooya, 2012	Polyglycerol dendrimers (PGDs)	Attenuation of ^19^F NMR signals with perfluorinated dendrimers	[91]
Tanaka et al., 2012	Water-soluble perfluorinated dendrimers	Evaluation of glutathione reductase (GR) activity by ^19^F NMR spectroscopy; GR enzymatic activity was determined from the increase in the size of the ^19^F NMR signals	[92]
Klemm et al., 2012	Esteramide (EA) dendrimer; PLLG2[Asp(COOH)PEO]_8_ Polylysine Dendrimer; Yb-TREN-Dendrimer; Dy-TREN-Dendrimer;	MRI contrast agents; these conjugates have relaxivities up to 374 mM^−1^ s^−1^ per dendrimer, high bioavailability, and low in vitro toxicity.	[93]
Chen et al., 2012	3rd-generation (G3) dendrimer	The integrin αvβ3 targeting ability of PEG-G3-(Gd-DTPA)6-(cRGD-DTPA)2 in vitro and in vivo was demonstrated	[94]
Klemm et al., 2012	Esteramide dendrimer (EA)	When covalently conjugated to a highly biocompatible esteramide dendrimer, T_2_ relaxation rates up to 52 mM^−1^ s^−1^ and T_1_ relaxation rates up to 31 mM^−1^ s^−1^ per gadolinium were observed under clinically relevant conditions	[95]
Tanaka et al., 2011	Perfluorinated dendrimers tethered on silica nanoparticles	Bimodal quantitative assay of enzymatic activity in (19) F NMR spectroscopy and fluorescence spectroscopy using a nanoparticle based molecular probe;	[96]
Nwe et al., 2010	4, 5, and 6 PAMAM dendrimer	This report presents the preparation and characterization of three [Gd-C-DOTA](-1)-dendrimer assemblies by way of analysis, NMRD spectroscopy, and photon correlation spectroscopy (PCS). Molar relaxivity measured at pH 7.4, 22 degrees C, and 3T (29.6, 49.8, and 89.1 mM^−1^ s^−1^ indicated the viability of conjugates as MRI contrast agents.	[97]
Tan et al., 2010	G2, G3	The peptide-targeted nanoglobular contrast agents showed greater contrast enhancement than the corresponding nontargeted agents in tumor at a dose of 0.03 mmol Gd/kg in female athymic mice bearing MDA-MB-231 human breast carcinoma xenografts.	[98]

**Table 3:** Use of dendrimers as contrast agents in in vivo studies.

Surname, Year	Type of Dendrimer/ Fluorination	Application and Results	Ref
Chen et al., 2020	5 poly(amidoamine) dendrimers, encapsulated gold nanoparticles, chelated gadolinium, and anti-human HER-2	Intravenous injection of this nanoparticle into mice with HER-2 positive breast tumors significantly increases the MRI signal intensity by ~20% and improves CT resolution and contrast by a factor of 2.	[100]
Zamani et al., 2020	Folic acid-conjugated G-3 99 m Tc-dendrimer	Breast cancer molecular imaging agent	[103]
Mekuria et al., 2018	G4.5 polyamidoamine (PAMAM) dendrimers	For the detection of a dual-channel carcinoma cell line (fluorescence/MR imaging) both in vitro	[105]
Zhang et al., 2017	Gadolinium-labeled dendrimer (FA-GCGLD)	Increasing the T1 contrast capacity in in vivo magnetic resonance angiography	[106]
Gonawala and Ali, 2017	G5 PAMAM dendrimer	For in vivo MRI studies in a preclinical animal model of glioma	[101]
Zhou et al., 2017	4th-generation zwitterionized biodegradable dendritic contrast agent (DCA)	As a deoditrinated biodegradable dendritic contrast agent to enhance the MRI of liver metastases	[102]
Mekuria et al., 2017	G4.5 dendrimers	As double (T_1_ and T_2_) contrast agents in magnetic resonance imaging	[107]
Filippi et al., 2017	Amphiphilic Janus-dendrimers (dendrimersomes); 3,5-C12-EG-(OH)4 dendrimer	As a contrast agent, T_1_ weighted enhancement in the tumor area	[108]
Xiong et al., 2016	Fourth-generation poly(propylene imine) (PPI) glycodendrimers	As a contrast agent for imaging the animal aorta, renal artery, kidneys, and bladder in in vivo studies	[109]
Li et al., 2016	Dendrimer nanoprobe labeled with cyclic arginine-glycine-aspartic acid pentapeptide (cRGDyK)	A contrast agent to differentiate the degree of liver fibrosis; the MR T1 signal weighted value increased in parallel with the severity of the liver fibrosis.	[110]
Filippi et al., 2015	Amphiphilic Janus-dendrimers (dendrimersomes)	Performance improvement in in vivo MRI studies in mice	[111]
Chen et al., 2015	Amine-terminated generation 5 poly(amidoamine) dendrimers	For targeted dual-mode computed tomography (CT)/magnetic resonance (MR) imaging of small tumors.	[112]
Yang et al., 2015	G5 dendrimer	Targeted magnetic resonance (MR) imaging of C6 glioma cells.	[113]
Nguyen et al., 2015	Manganese (Mn) G8 dendrimers	For imaging atherosclerotic lesions with 3 Tesla MRI.	[99]
Li et al., 2013	Amine-terminated generation 5 poly(amidoamine) dendrimers	A contrast agent for magnetic resonance (MR)/computed tomography (CT) imaging of breast cancer cells in vitro	[114]
Chen et al., 2013	Amine-terminated generation 5 poly(amidoamine) dendrimers (G5.NH2)	For imaging tumors in CT and MRI, it shows a high intensity of radiation suppression and improved MRI contrast.	[115]
Mohamadi et al., 2013	Dendrimer G1	The uptake of the drug into the liver hepatocellular cell line and the drug cytotoxicity were evaluated. It also increases the relaxivity of the tissue and enhances the MR images contrast.	[104]
Ye et al., 2013	2nd-generation dendrimer (G2)	Biodegradable dendritic contrast agent (DCA) (FA-PEG-G2-DTPA-Gd) was prepared from a polyester dendrimer conjugated with gadolinium (Gd) chelates and PEG chains with distal folic acid. The MRI contrasted by FA-PEG-G2-DTPA-Gd outlined the inoculated tumor more clearly and had much higher contrast enhancement for a much longer time than Magnevist.	[116]
Wen et al., 2013	Amine-terminated generation five poly(amidoamine) dendrimers (G5.NH_2_)	Were used as templates to synthesize gold nanoparticles (AuNPs). With the coexistence of the two radiodense imaging elements of AuNPs and Gd(III) within one NP system, the formed Gd-Au DENPs display both r_1_ relaxivity for the MR imaging mode and the X-ray attenuation property for CT imaging mode, which enables CT/MR dual mode imaging of the heart, liver, kidneys, and bladder of rats or mice.	[117]
Andolina et al., 2012	Esteramide dendrimer (EA)	DyN1-EA had the largest ionic T(1) relaxivity, 7.60 mM^−1^ s^−1^, while YbN3-EA had the largest ionic T(2) relaxivity, with an NIR quantum yield of 0.17 % when evaluated in mouse serum. This is the first Yb(III) bimodal NIR/T(2) MRI contrast agent of its kind that has been evaluated.	[118]
Huang et al., 2012	Individual dendrimers	Biodegradable DNCs were prepared with polydisulfide linkages between the individual dendrimers. DNCs possessed a circulation half-life of >1.6 h in mice and produced significant contrast enhancement in the abdominal aorta and kidneys for as long as 4 h.	[119]
Lim et al., 2012	Dendrimers generation 5 and 3 (G3 and G5) and four gadolinium (Gd)-based macromolecular contrast agents, G3-(Gd-DOTA)(24), G5-(Gd-DOTA)(96), G3-(Gd-DTPA)(24), and G5-(Gd-DTPA)(96),	These triazine dendrimer-based MRI contrast agents exhibit several promising features such as high in vivo r1 relaxivity, desirable pharmacokinetics, and well-defined structure.	[120]
Nwe et al., 2012	Dendrimer G4 and G5, Gd-DOTA (G4SS30, G5SS58), respectively	The in vitro molar relaxivity of the Ab-(G4S15)(4) conjugate measured at pH 7.4, 22 °C, and 3T showed a moderate increase in relaxivity as compared to Magnevist (6.7 vs. 4.0 mM^−1^ s^−1^, while the Ab-(G5S29)(4) conjugate was two-fold higher (9.1 vs. 4.0 mM^−1^ s^−1^.	[121]
Luo et al., 2011	Third generation (G3) peptide dendrimers; L-lysine-based dendrimer	In vivo studies have shown that the mPEGylated Gd(III)-based dendrimer provided much higher signal intensity enhancement (SI) in mouse kidneys, especially at 60 min post-injection, with 54.8% relatively enhanced SI.	[122]
Kojima et al., 2011	PAMAM dendrimers (generations 4 and 5; G4 and G5)	Surface-PEGylated Gd-PAMAM dendrimers showed decreased plasma clearance and prolonged retention in the blood pool. Shorter PEG, higher generation conjugates led to higher relaxivity.	[123]
Nwe et al., 2010	PAMAM dendrimer generation 4 (G4 dendrimer), gadolinium-dendrimer conjugates of derivatized acyclic Diethylenetriamine-N,N′,N′,N″, N″-pentaacetic acid (1B4M-DTPA) and macrocyclic 1,4,7,10-tetraazacyclododecane-N,N′,N″,N‴-tetraacetic acid (C-DOTA).	The macrocyclic-based agent is the more suitable agent for in vivo use for these reasons combined with kinetics. Inertness is associated with the Gd(III) DOTA complex stability properties.	[124]

## Data Availability

The data presented in this study are available on request from the corresponding author.

## Sample Availability

Samples of the compounds are available from the authors.
